# A Simple LC–MS Method for the Quantitation of Alkaloids in Endophyte-Infected Perennial Ryegrass

**DOI:** 10.3390/toxins11110649

**Published:** 2019-11-07

**Authors:** Simone Vassiliadis, Aaron C. Elkins, Priyanka Reddy, Kathryn M. Guthridge, German C. Spangenberg, Simone J. Rochfort

**Affiliations:** 1 Agriculture Victoria, AgriBio Centre for AgriBioscience, Bundoora, VIC 3083, Australia; aaron.elkins@agriculture.vic.gov.au (A.C.E.); priyanka.reddy@agriculture.vic.gov.au (P.R.); kathryn.guthridge@agriculture.vic.gov.au (K.M.G.); german.spangenberg@agriculture.vic.gov.au (G.C.S.); simone.rochfort@agriculture.vic.gov.au (S.J.R.); 2 School of Applied Systems Biology, La Trobe University, Bundoora, VIC 3083, Australia

**Keywords:** *Epichloë* endophyte, *Lolium perenne* L., ryegrass, alkaloid, mycotoxins, quantitation, liquid chromatography–mass spectrometry

## Abstract

The rapid identification and quantitation of alkaloids produced by *Epichloë* endophyte-infected pasture grass is important for the agricultural industry. Beneficial alkaloids, such as peramine, provide the grass with enhanced insect protection. Conversely, ergovaline and lolitrem B can negatively impact livestock. Currently, a single validated method to measure these combined alkaloids in planta does not exist. Here, a simple two-step extraction method was developed for *Epichloë*-infected perennial ryegrass (*Lolium perenne* L.). Peramine, ergovaline and lolitrem B were quantified using liquid chromatography–mass spectrometry (LC–MS). Alkaloid linearity, limit of detection (LOD), limit of quantitation (LOQ), accuracy, precision, selectivity, recovery, matrix effect and robustness were all established. The validated method was applied to eight different ryegrass-endophyte symbiota. Robustness was established by comparing quantitation results across two additional instruments; a triple quadruple mass spectrometer (QQQ MS) and by fluorescence detection (FLD). Quantitation results were similar across all three instruments, indicating good reproducibility. LOQ values ranged from 0.8 ng/mL to 6 ng/mL, approximately one hundred times lower than those established by previous work using FLD (for ergovaline and lolitrem B), and LC–MS (for peramine). This work provides the first highly sensitive quantitative LC–MS method for the accurate and reproducible quantitation of important endophyte-derived alkaloids.

## 1. Introduction

Grasses form an economically important component of forage and turf, accounting for a quarter of the world’s vegetation [1]. In Australia, perennial ryegrass (*Lolium perenne* L.) is the most utilized pasture grass on dairy farms [2]. In pastoral agriculture, perennial ryegrass is often infected with asexual *Epichloë* endophytes. In asymptomatic interactions, endophytes are transmitted vertically from host grass to seed [3,4], forming symbiotic relationships with their host. Systemic colonization then occurs throughout the aerial portions of the grass’ tissues [5]. The host plant provides the endophyte with shelter, nutrition and the ability to reproduce [5,6], while the endophyte increases the competitive ability of the grass; and infected plants can exhibit enhanced growth, vigor, reproduction and persistence [7]. The endophyte also benefits the plant by providing increased tolerance to abiotic stresses, such as drought and mineral stress [8,9]. Additionally, biotic protection has been associated with the production of endophyte-derived alkaloids, a class of secondary metabolites which confer resistance against herbivores and pathogens [10,11].

The consumption of endophyte-infected grasses can be detrimental to the health of livestock. Ergovaline (an ergot alkaloid) and lolitrem B (an indole-diterpene alkaloid) are toxic to grazing mammals. Ergovaline causes fescue toxicosis and lolitrem B, a tremorgenic neurotoxin, causes ryegrass staggers disease [12,13,14,15]. A loss of 13% of steers, bulls and pregnant beef cows occurred after being fed a diet supplemented with perennial ryegrass pellets. Screening of the pellets showed ergovaline concentrations between 800 and 1100 ppb [16]. Additional case studies showed that 47% to 54% of cattle consuming perennial ryegrass straw with lolitrem B concentrations at 2574 parts per billion (ppb) and 2017 ppb developed ryegrass staggers, whilst those consuming levels as low as 1400 ppb did not [16,17,18]. Conversely, a beneficial alkaloid, peramine (a pyrrolopyrazine alkaloid) is a potent insecticide [19,20,21,22]. Peramine, although highly active against insect pests, has no known toxic impacts on grazing livestock. Production and monitoring of both beneficial and detrimental alkaloids are, therefore, of economic importance within the pastoral farming industry.

Different grass–endophyte associations produce different combinations of alkaloids. Type and concentration can be influenced by plant genotype, endophyte genotype, tissue type and growing season [9,19]. Therefore, it is importance to select grass–endophyte associations which minimally impact livestock, yet still produce beneficial anti-insect compounds. The ability to screen alkaloid content in grass–endophyte associations is required for the determination of pasture toxicity and for the evaluation of novel associations prior to commercialization. Early methods employed high performance liquid chromatography (HPLC) with ultraviolet detection for the quantitation of lolitrem B from grasses [23]. However, interference by other compounds in the plant matrix masked the detection of low-level lolitrem B, and a more robust method using HPLC with a fluorescence detector (FLD) was implemented [24,25]. Similar HPLC methodologies have been used for the quantitation of ergovaline [25,26,27,28,29,30,31] and peramine [30,32], whilst gas chromatography coupled to mass spectrometry (CG–MS) has been used to analyze other endophyte-derived alkaloids such as lolines [33,34].

Often, multiple extraction and analytical methods are employed to analyze alkaloids in grasses, [25,30,32]. For example, Moore et al. used different extraction and analytical methods for the analysis of alkaloids from *Epichloë*-infected ryegrass species [35]. Such methods included HPLC with FLD to quantify both ergovaline and lolitrem B, and liquid chromatography coupled to mass spectroscopy (LC–MS) to quantify peramine [35,36]. Near Infrared Reflectance Spectroscopy (NIRS) has recently been used for the quantitative analysis of peramine, lolitrem B and ergovaline from perennial ryegrass [37]. Although NIRS offers a fast, clean and cheap method for forage quality control, it is not at all sensitive enough to detect accurate alkaloid concentrations. The advancement of mass spectrometry technologies has led to additional methods for the simultaneous extraction and quantitation of mycotoxins [38]. Currently, LC–MS is the most widely used method of analysis [14,36], specifically for lolitrem B [39,40], ergovaline [41,42] and peramine [43].

Recent work has utilized LC–MS for the quantitation of peramine, lolitrem B and ergovaline from endophyte-infected grass [44,45]; however, the methods have limited detail regarding validation procedures as described by Peters et al. [46]. Therefore, the objective of this study was to develop a simple method to extract and quantitate important alkaloids from the shoots of *Epichloë*-infected perennial ryegrass plants. This work reports the development and validation of a LC–MS method for the quantitative analysis of peramine, ergovaline and lolitrem B. Validation of the method was tested across two additional instruments using a suite of different perennial ryegrass–endophyte associations. The purpose of this was to compare the LC–MS method developed in this study to current industry-standard methods of quantitation, using a triple quadrupole MS system (QQQ MS) for peramine, lolitrem B and ergovaline [44,45], and by HPLC with FLD for ergovaline only [32,35]. To our knowledge, this is the first highly sensitive, quantitative method for the high-throughput analysis of endophyte-derived alkaloids in perennial ryegrass using a single validated LC–MS run.

## 2. Results

### 2.1. Method Performance

**Figure 1:**
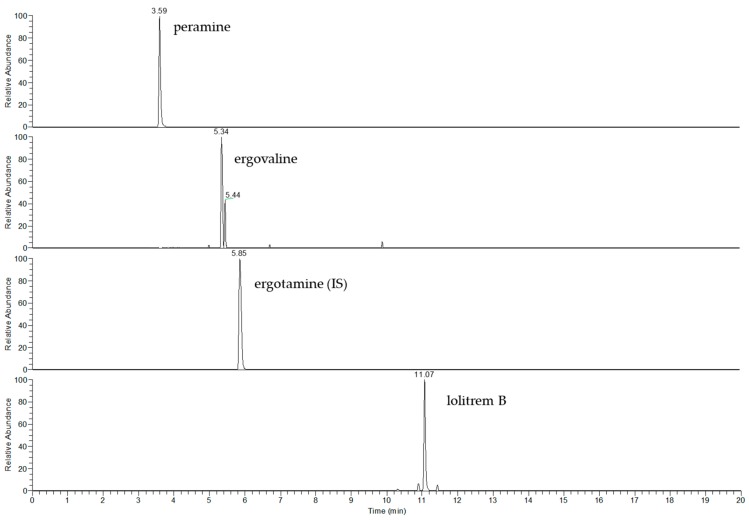
Extracted ion chromatogram (EIC) of peramine, ergovaline, ergotamine and lolitrem B in perennial ryegrass with standard toxic endophyte (E+). Ergotamine was used as an internal standard (IS) for ergovaline.

For all preliminary LC–MS method validation experiments, a Thermo Scientific Q Exactive Plus mass spectrometer (QE MS) (Bremen, Germany) was used. The accurate mass for each alkaloid was compared with analytical standards, except for ergovaline which was not available at the time of the study (Table 1). Hence, ergotamine, an alkaloid with structural similarity, was used to quantify ergovaline. The extracted ion chromatogram (EIC) of a perennial ryegrass sample with standard toxic endophyte (SE) profile (E+) is illustrated in Figure 1. The more-polar alkaloids, including peramine, ergovaline and ergotamine, eluted at 3.59, 5.34 and 5.85 min, respectively. Two chromatographic peaks were observed from the extracted ion chromatogram of ergovaline. This isomer, likely ergovalinine, was also integrated for quantitation purposes [2,30]. The more-hydrophobic alkaloid, lolitrem B, eluted at 11.07 min.

### 2.2. LOD, LOQ and Linearity

The limit of detection (LOD) and limit of quantitation (LOQ) are detailed in Table 1. The method showed good sensitivity for detecting peramine and ergotamine/ergovaline, with LOD and LOQ values determined at 0.2 and 0.8 ng/mL, respectively for both compounds (Table 1). The linear range was 0.8 to 1593.9 ng/mL (peramine) and 0.8–1684 ng/mL (ergotamine). The method was less sensitive for lolitrem B, with a LOD value of 1.8 ng/mL and linear range between 6 and 2400 ng/mL. All *R*^2^ values were above 0.99.

### 2.3. Accuracy (Bias), Precision and Selectivity

Accuracy (bias) and precision (% relative standard deviations, RSD) were established by comparing three different standard preparations at low, medium and high concentrations relative to the calibration curves (Table 2). All standards were below 3.5% RSD, with all measurements determined at less than 1% of the expected concentration. Selectivity of the method was assessed by visually inspecting the chromatograms of endophyte-free (E−) plants to determine potential interferences which may impact the sample matrix. No co-eluting peaks were observed in the EICs of the alkaloids. However, closer inspection of the mass spectral data shows the co-elution of many other ions in E− plants when compared to those spiked at low concentrations. This is illustrated by a representative perennial ryegrass E− sample spiked with peramine at approximately 3.59 min (Supplementary Figure S1). Compounds from a complex plant matrix can interfere with the identification of the observed mass peak in an EIC, thus, it is imperative that the analysis of the plant matrix is considered.

### 2.4. Extraction of Alkaloids

The optimal protocol for the extraction of alkaloids from E+ plants was explored (Table 3). The first method (‘method 1’) assessed the amount of analyte removed from the plant after five extractions. This was compared with an already established protocol (‘method 2’) which utilized two combined extractions and involved a drying/reconstitution step [43].

Two extractions consistently removed most of the analytes from the sample. Although very small amounts of peramine and lolitrem B were still detected in extractions three to five, these values were below, or close to, the limit of quantitation. When summed, peramine concentrations from the first two extracts using ‘method 1’ exceeded those of ‘method 2’ (39.5 ppm and 33.2 ppm, respectively), and this was similar for lolitrem B (15.3 ppm and 14.3 ppm, respectively). Endogenous ergovaline was present at very low concentrations and only one extraction was required, however, quantitative results from both methods were consistent (0.1 ppm). Precision of each method was established by RSD values between 0.45–8.3% for peramine, 2–2.2% for ergovaline, and 1.2–7.5% for lolitrem B (Table 3).

### 2.5. Matrix Effect and Recovery

Neat standards, pre- and post-spiked perennial ryegrass samples at low, medium and high concentrations were employed to assess the matrix effect (ME) and determine the recovery (RE) of alkaloids from samples (Table 4). A matrix effect was observed in the form of ion suppression, indicated by ME values <100%. Peramine and lolitrem B showed moderate ion suppression with ME averages of 67–81% and 67–88%, respectively, observed across the E− and E+ quality control (QC) samples. Less matrix effect was observed for ergotamine, with values ranging between 73–77%. Recoveries of spiked samples were relatively high, ranging from 77–88% for peramine, 85–87% for ergotamine and 80–90% for lolitrem B. These results were consistent for analysis of both of E− and E+ plants. Recovery rates were reproducible as indicated by low RSD values: neat standards, <4%; pre-spikes, <7% and post spikes, <5% (Supplementary Table S1).

### 2.6. Method Robustness and Application

Robustness of the method was tested on commercially available glasshouse-grown perennial ryegrass plants infected with a suite of different endophyte strains. For comparative purposes, the extracts were injected across three analytical platforms. All alkaloids were quantified using two LC–MS systems, the QE MS and the QQQ MS. Ergovaline was also analyzed using an established HPLC-FLD method (Agilent 1100 Series HPLC System) (Agilent, Santa Clara, CA, USA).

**Figure 2:**
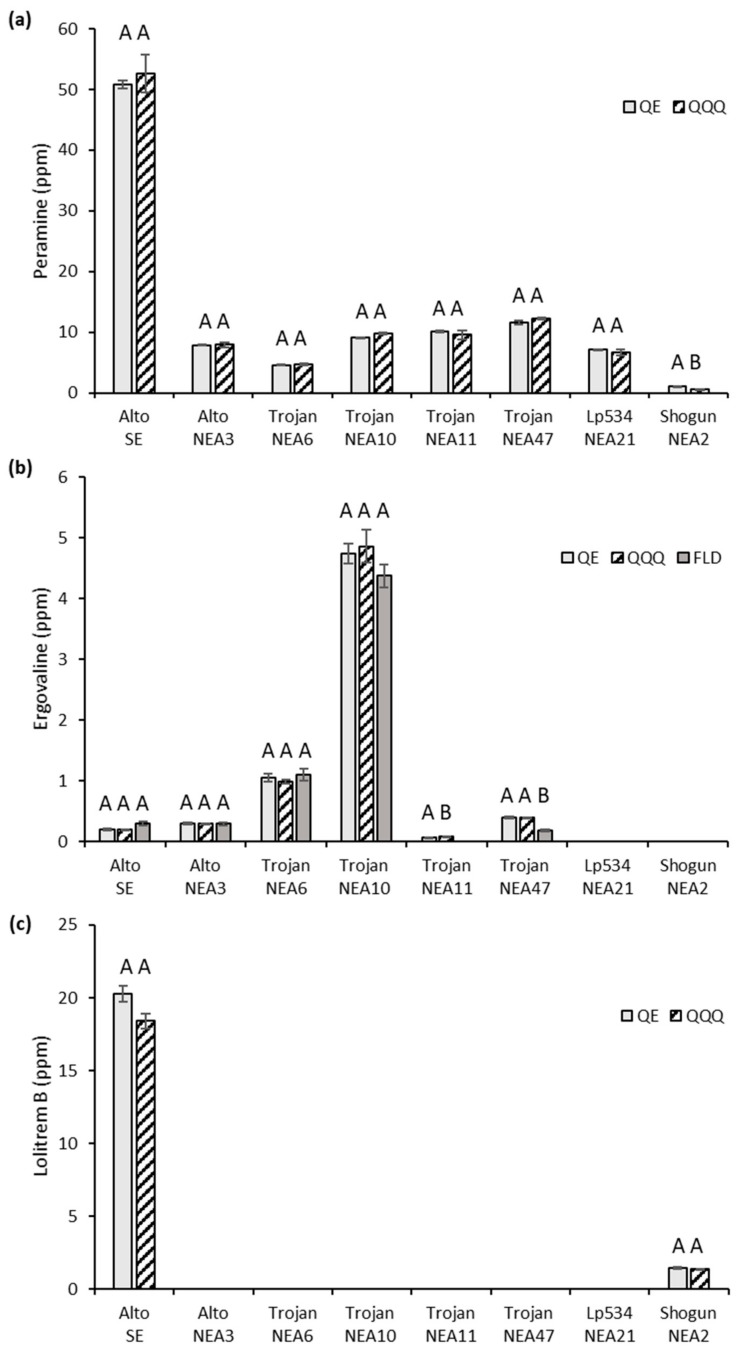
Comparison of mean alkaloid concentrations (parts per million, ppm; mg/kg) in eight different glasshouse-grown ryegrass–endophyte associations using the Thermo Q Exactive Plus (QE) and Agilent 6460C Triple Quadruple (QQQ) mass spectrometers, and the Agilent 1100 fluorescence detector (FLD): (**a**) peramine, (**b**) ergovaline and (**c**) lolitrem B. No alkaloids were detected in Trojan-WE (omitted). Bars represent the standard error of the mean (SEM). Bars with different letters are significantly different when compared to the QE (*t*-test, *p* < 0.05).

As expected, alkaloids were not detected in perennial ryegrass without endophyte (Trojan-WE) and the abundance of alkaloids in each of the associations differed greatly (Figure 2). Alto-SE produced the highest amount of peramine and lolitrem B (~50 and 20 ppm, respectively), whilst Trojan-NEA10 produced the greatest amount of ergovaline (~5 ppm). Quantitation values from the QQQ MS compared to the QE MS showed no significant difference except for those with very low alkaloid concentrations of less than 1 ppm, i.e., peramine levels in Shogun-NEA2 (difference of 47%, *p* = 0.0005) and ergovaline levels in Trojan-NEA11 (difference of 41%, *p* = 0.02) (Supplementary Table S2). Ergovaline results were similar when detected by the FLD compared to the QE MS; no significant differences between the quantitative values were observed, except for Trojan-NEA47 in which the FLD detected half the amount of ergovaline (0.39 ppm determined by the MS systems, and 0.18 ppm determined by the FLD instrument, *p* = 0.0004) (Supplementary Table S2).

## 3. Discussion

*Epichloë*-infected perennial ryegrass is used as pasture forage for livestock. *Epichloë* endophyte strains are known to produce alkaloids as a protective measure against insect and animal herbivory. Of these, peramine is beneficial, as it deters insect herbivory with no impact to animal health. Others, including ergovaline and lolitrem B, can be detrimental to livestock, inducing toxicosis and ryegrass staggers. The concentration of toxic alkaloids varies and is often influenced by factors such as season. Hence, a simple yet fast analytical method is required for the analysis of alkaloids to mitigate and/or diagnose the source of disease outbreaks. Moreover, a method to screen novel ryegrass–endophyte associations is of interest for the agricultural industry.

This study validates a simple extraction and analytical method for the quantitation of the three important endophyte-derived alkaloids in perennial ryegrass using LC–MS. We modified and combined recently published methods, aimed at quantifying peramine [43] and lolitrem B [39]. The new method was then tested using a suite of *Epichloë*-infected perennial ryegrass samples with differing endophyte-derived alkaloid profiles. Robustness of the method was determined by employing alternative industry-standard instruments such as a triple quadrupole MS (QQQ MS, for all alkaloids) [44] and HPLC-FLD (for ergovaline only) [35].

For all LC–MS experiments, a linear gradient ranging from 2–100% mobile phase B (acetonitrile with 0.1% formic acid) over 15 min with a reverse-phase C18 was applied to assess endophyte-derived alkaloids in planta. Elution times for peramine and lolitrem B were consistent with previous work [39,43]. Quantitation was performed using external standards for peramine and lolitrem B. An analytical standard was not available for ergovaline, so quantitation was performed using another ergopeptine, ergotamine, which is not naturally produced by the endophyte [30]. Ergot alkaloids, including ergotamine, are susceptible to epimerization [14,47,48]. The extent of epimerization depends on many variables including light, heat and the amount of time the compound has been dissolved in solution [47,49]. Although samples were kept in the dark and immediately run after extraction, isomerization was still evident; two co-eluting peaks were observed across all instruments. This is not uncommon, and research suggests the stability of ergovaline is compromised only 24 h after harvesting plant tissues [47]. It is, therefore, likely that ergovaline was isomerized to ergovalinine [30]. To account for this, the peak areas of ergovaline and the isomer were included into the calculation for compound quantitation [2,30].

Performance characteristics of the method were similar across all the instruments tested. The LOD and LOQ of peramine and ergovaline/ergotamine was below 1 ng/mL, whilst lolitrem B was below 6 ng/mL (from both MS instruments). These limits were much lower than those documented by Fuchs et al., in which alkaloids were detected at 5 ng/mL [50]. Analysis of ergovaline/ergotamine by FLD was less sensitive, with LOD and LOQ values estimated below 2 ng/mL and 8 ng/mL, respectively. All reported values in this study are, however, in line with current industry ‘safe feed’ threshold levels for toxicosis, whereby ergovaline levels are 500–800 ppb (ppb, equal to ng/mL) for sheep and 300–500 ppb for cattle, and lolitrem B levels are 1800–2000 ppb for both sheep and cattle (dependent on weather) [16,17].

Although most of the quantitation values were calculated from linear calibration curves, oversaturation of peramine was observed using the QQQ MS instrument, as illustrated by a plateau of the higher calibration levels. Hence, a power curve was used to calculate the *R*^2^ value (Supplementary Table S3). This suggests that analysis of samples with high peramine levels may need careful consideration as values may fall outside of the calibration curve. To compensate for this, such samples need to be diluted. This would be time consuming and costly, and, therefore, not appropriate for high-throughput analysis of peramine from perennial ryegrass. Nonetheless, the overall method established using the QE MS system was highly accurate, as determined by the mean difference between the theoretical result and actual result of the alkaloid standards, and results were well-within the acceptance criteria of 15% from the reference value [51]. Similarly, precision of the method was also well within the acceptable value of 15% RSD [46,51].

Extraction of alkaloids from the plant matrix was assessed by comparison of two methods with single or multiple extractions, and with a drying/reconstitution step. The purpose of this was to improve a previously established method [43]. Results illustrated that our simple two-step extraction was adequate for the removal of the three alkaloids targeted in this study. Very small concentrations of peramine and lolitrem B remained after the first two extractions, however, the quantitative results were below the LOQ and the additional extractions were deemed unnecessary. Further, total alkaloid concentrations were the same when comparing the two extraction methods. Removal of the reconstitution step in results in less handling time, allowing the two-step extraction process to take no more than 10 min (vortex and sonicating time). This is faster compared to other published methods in which the process has been reported to take up to one hour [30,32,52].

Selectivity of blank matrix samples (E−) were established by visual inspection to check for interfering signals [46]. Although no endogenous endophyte-derived alkaloids were observed within the E− plants, analysis of the mass spectral data indicated that the plant matrix is quite complex, and co-elution of other compounds at a given retention time is inevitable. Such results stress the importance of assessing plant matrix effects and recovery rates of alkaloids from QC samples.

Generally, recovery experiments are calculated by comparing an analyte’s response after sample-handling with that of a solution containing the analyte at the same concentration [46]. Unfortunately, some studies may not consider the impact of the sample matrix when establishing recovery protocols, and external standards are instead used for comparison. Therefore, guidelines suggest that recoveries should be established by use of a pre- and post-extraction spike to account for matrix interferences; and further, recovery rates should be greater than 50% [46].

Relatively high recovery rates of the spiked alkaloid standards (77–90%) were observed in this study, irrespective of the sample (E− or E+ plants). This indicates that most of the analyte can be recovered; a consistent outcome observed from preliminary studies in which peramine and lolitrem B profiles were still detected at low concentrations after two extractions. The recovery of loline-type alkaloids has been documented between 87–126% from spiked grass samples, irrespective of the extraction solvent (isopropanol/water or methanol/water) or method (shaking or sonicating) [52]. Nevertheless, results reported here are comparable to other studies which recovered 77–86% ergotamine from grass tillers, and this also appears to be consistent with other reported recovery results [26,28,30].

Indeed, recovery rates of less than 100% may suggest interference from the plant matrix. Complementary analysis of the sample matrix allows for determination of the potential ion suppression or enhancement caused by co-eluting compounds. Discussed above, the complexity of the sample matrix was established, and it is not surprising that suppression of the targeted ions was observed (i.e., ME results were determined at <100%). To compensate for this, it is suggested that the plant matrix should be considered when developing further quantitative alkaloid studies. Additionally, quantitative studies should correct final values for recovery rates and employ matrix-matched standards to build calibration curves.

Considering the above, robustness of the method was established using a suite of perennial ryegrass plants hosting a suite of different commercial and non-commercial (NEA10, NEA11 [53], and NEA21 [54]) endophytes. The extracts were injected across three analytical platforms. All alkaloids were quantified using two LC–MS systems, the QE MS and the QQQ MS. Ergovaline was also analyzed using the FLD. The purpose of this was to compare the quantitative results from our LC–MS method to similar methods [32,35,44,45]. There was good agreement between the results obtained across the three different techniques, except for samples with very low alkaloid concentrations. Shogun-NEA2 and Trojan-NEA11 showed significant differences in the levels of peramine and ergovaline, respectively, between the QE MS and QQQ MS. Likewise, ergovaline levels in Trojan-NEA47 showed significant differences between the FLD and QE MS results. In all instances, these concentration values were approximately 40–50% higher when detected by the QE MS system. The methods reported here established low LOQ values ranging from 0.8 ng/mL to 6 ng/mL, which is approximately one hundred times lower than those established by previous work using FLD (for ergovaline and lolitrem B), and LC–MS (for peramine) [35]. It should be noted that growth conditions for these plants were such that they do not necessarily represent actual field levels with our glasshouse grown material having almost double the level of ergovaline compared to the field grown plants that we have analyzed previously [55]. Nonetheless, results highlight the vast differences between alkaloid concentrations in endophyte infected perennial ryegrass plants, and therefore, the accurate detection and quantitation of alkaloids at very low or very high concentrations is of equal importance.

## 4. Conclusions

The alkaloid extraction method developed here is simple yet robust. Use of LC–MS for quantitation gives greater sensitivity and provides accurate and precise results. The method is fast and can, therefore, be used for high-throughput screening of endophyte-derived alkaloids from perennial ryegrass. It may also be adapted for the future analysis of alkaloids from other important forage grasses such as tall fescue, or animal tissues whereby very low amounts of a mycotoxin may be significant. Finally, acquisition of all MS data may also provide the added benefit of future biochemical pathway analysis (via untargeted MS/MS metabolomics) which is required to supplement the design of novel grass–endophyte associations.

## 5. Materials and Methods

### 5.1. Chemicals

All extraction and mobile-phase solvents were of HPLC grade. Methanol (≥99.9% pure), acetonitrile with 0.1% formic acid (≥98.5% pure) and water with 0.1% formic acid were purchased from Fisher Chemical (Fair Lawn, NJ, USA). Ammonium formate (≥99.0% pure) was purchased from Sigma-Aldrich (St. Louis, MO, USA). The alkaloid standards used in the study were peramine nitrate (BDG Synthesis, Wellington, New Zealand), ergotamine D-tartrate (≥97.0% pure; Sigma-Aldrich, St. Louis, MO, USA) and lolitrem B isolated from perennial ryegrass seeds by Reddy et al. [39].

### 5.2. Plant Material

Perennial ryegrass (*Lolium perenne* L.) seeds were supplied by Barenbrug Agriseeds (Christchurch, New Zealand). Seeds were planted in 42-cell plant trays filled with potting mix containing seed-raising mix (30 L) (Van Schaik’s Biogro, Dandenong South, Victoria, Australia) mixed with fine vermiculite (0.9 L), fine perlite (0.6 L), micro nutricote (25 g), water-holding granules (20 g) and trace elements (2.5 g). Germination and growth occurred under glasshouse conditions (average of 25 °C, natural lighting with a 16 h photoperiod and approximately 60% relative humidity). Confirmed endophyte-positive seedlings were transplanted into 8 × 8cm pots and maintained under glasshouse conditions.

### 5.3. Sample Preparation

Mature plants were harvested approximately 5 cm from the base of the plant with clean scissors, placed into paper bags and immediately transferred to −80°C. Samples were freeze-dried (Christ Alpha 1–4 LD plus) for approximately 48 h then ground to a fine powder using a Genogrinder 2010 at a frequency 1500 rpm for 2 min. QC perennial ryegrass samples with standard toxic endophyte (E+) and without endophyte (E−) were harvested from glasshouse-maintained plants (growth conditions described above). The QC plants were used to compare the presence or absence of targeted compounds.

### 5.4. Sample Extraction

Two extraction methods were investigated using pooled E+ QC samples (20mg ± 0.2mg). The first extraction method (‘method one’) was designed to observe the total amount of analyte removed from the plant matrix after multiple extractions. Ground plant material was extracted with 1 mL of 80% methanol (methanol and milli-Q water, 80:20, *v*:*v*). Samples were vortexed for 5 min (Ratek multi tube vortex mixer, MTV1, Boronia, Victoria, Australia), sonicated for 5 min (SoniClean, 250TD, Thebarton, South Australia) and centrifuged for 5 min at 161,000× *g*, 21 °C (Eppendorf, 5415D, Hamburg, Germany). The supernatant was transferred to an empty tube. The pellet was re-extracted four more times and the supernatant transferred to an empty tube each time. A 150 µL aliquot from each extract was then transferred to individual 2 mL HPLC vials with 200 µL inserts (i.e., five vials per extract).

‘Method two’ was replicated from an established method aimed at quantifying peramine [43]. This method employed a drying and reconstitution step to re-concentrate the sample 10-fold. Here, ground plant material was extracted twice as above, and the supernatants were combined into the same tube. These were dried using a SpeedVac Concentrator (Thermo Fisher Scientific, Savant SPD 2010) at room temperature for approximately 16 h. The extracts were reconstituted in 200 µL of 80% methanol, and 150 µL was transferred to individual 2 mL HPLC vials with 200 µL inserts. Each test was completed using triplicate sample extracts.

### 5.5. Method Validation

Method validation characteristics were evaluated using international guidelines (Peters et al. [46]). Validation parameters included linearity, LOD, LOQ, accuracy (bias), precision, selectivity, matrix effect (ME) and recovery (RE).

The linear range encompasses the points between the LOQ and the highest concentration detected by the instrument, whilst still maintaining linearity (i.e., an *R*^2^ value equal to or greater than 0.99). The linear range for individual alkaloids was determined using a mixed standard whereby a series of nine dilutions were prepared from within a range of 0.8–1593.9 ng/mL (peramine), 0.8–1684 ng/mL (ergotamine) and 6.0–2400 ng/mL (lolitrem B). Concentrations were selected through preliminary testing in solvent (80% methanol). Linear regression models were created by repeated analysis (*n* = 5) of the chemical standards.

The LOD and LOQ was estimated by measuring the peak-to-peak signal-to-noise ratios (S/N) whereby S/N > 3 and S/N > 10, respectively [46]. Matrix-matched standards using E- QC samples were used for all other quantitation work after proving linearity.

Accuracy (bias) and precision were assessed by analyzing three different standard concentrations; one close to the LOQ (Low), one intermediate (Med), and one close to the upper limit of the curve (High) (i.e., peramine at 0.79, 79.7 and 796.9 ng/mL; ergotamine at 8.4, 84.2 and 842.0 ng/mL; and lolitrem B at 12, 120 and 1200 ng/mL). Duplicate injections of the standards were analyzed eight times. Accuracy (bias) was calculated based on the percent deviation from the expected concentration value, whilst precision was calculated based on the percent relative standard deviation (% RSD) of the analyte peak response.

Selectivity of the method was assessed by visually inspecting the chromatograms of non-spiked and spiked E− samples. The presence of interfering or co-eluting compounds from the plant matrix were established.

The potential matrix effect (ME) was established by comparing the response of post-extraction spiked E− samples with the response of the neat standard at the same concentration (Low, Med and High standards). Ion suppression was determined at ME <100%, whilst enhancement was determined at ME >100%. Recovery (RE) of the analytes were established by comparing the response of analytes from pre-extraction spiked samples to the post-extraction spiked samples (E−). These validation parameters were also applied to E+ QC samples as above, however, the presence of endogenous alkaloids (peramine and lolitrem B) identified in non-spiked samples (NS) were subtracted from the equation. Full recovery was determined at RE = 100%. The equations used for ME and RE are given below:

$$ME=\frac{post-extraction\;spike}{neat\;standard}\;\times 100\%\;RE=\frac{pre-extraction\;spike}{post-extraction\;spike}\;\times 100\%$$

### 5.6. Analytical Instruments and Conditions

All ryegrass samples, including those used for the validation tests, were analyzed using a Thermo Scientific Vanquish ultra-high-performance liquid chromatography (UHPLC) system (Thermo Fischer Scientific, Bremen, Germany) coupled to a Thermo Fisher Q Exactive Plus mass spectrometer (QE MS) (Waltham, MA, USA; Thermo, Bremen, Germany). The LC–MS methods were modified from an established method aimed at quantifying peramine [43]. Analytes were separated on a 100 mm × 2.1 mm Thermo Hypersil Gold, 1.9 µm HPLC column with a gradient mobile phase of A, 0.1% formic acid in water and B, 0.1% formic acid in acetonitrile. The column compartment was maintained at 30 °C. The initial conditions were 2% B before initiating a linear gradient to 100% B over 11 min, and this was maintained for 4 min before returning to the initial gradient conditions at a flow of 0.3 mL/min (total run time of 20 min) [43]. The extracts (3 µL) were injected onto the system and analyzed in positive electrospray ionization (ESI) mode over a mass range of 80–1200 *m*/*z*. Instrument resolution was set at 35,000, normalized collision energy was 30 V and the maximum ion time was 200 milliseconds. The source heater temperature was maintained at 310 °C and the heated capillary was maintained at 320 °C. The sheath, auxiliary and sweep gases (N_2_) were 28, 15 and 4 units, respectively. The spray voltage was set at 3.6 kV. Prior to data acquisition, the system was calibrated with Pierce^®^ LTQ Velos ESI Positive Ion Calibration Solution (Thermo Scientific, product no. 88323).

For preliminary assessments, the molecular ions were extracted from the full scan chromatograms and the peak areas were integrated using Thermo Xcalibur Qual Browser v.3.0.63 (Thermo Scientific). The alkaloids were then quantitated using LCquan v2.7.0.21 (Thermo Scientific). Typical mass accuracy for the alkaloids was 3 ppm or better. Peramine and lolitrem B were matched to analytical standards, whilst ergotamine was used to quantify ergovaline.

Robustness of the method was established using two additional analytical instruments. These experiments were performed at the same time using different UHPLC systems on different columns. Analytical standards were prepared in plant matrix (E−) using the full calibration range.

The first instrument was an Agilent 1290 UHPLC system coupled to an Agilent 6460C Triple Quadruple mass spectrometer (QQQ MS) (Agilent, Santa Clara, CA, USA). Data was acquired using an Agilent Jet Stream ESI source in positive ionization mode. All UHPLC parameters including the column, mobile phase and instrument conditions were as previously described above. For the QQQ MS conditions, the sheath gas temperature was set to 300 °C, the sheath gas flow was 5 L/min, the nebulizer pressure was 30 psi, and the capillary voltage was 4200 V. Dynamic multiple reaction monitoring (MRM) mode parameters for the targeted compounds were optimized through Agilent optimizer software (MassHunter Optimizer), including the MRM transitions, the collision energy (15 V for peramine, 30 V for ergotamine/ergovaline and lolitrem B), the fragmentor voltage (135 V), the dwell time (200 ms) and the cell accelerator voltage (7 V). Data was processed by MassHunter qualitative analysis software v.B.06.00 (Agilent, Santa Clara, CA, USA).

Ergovaline was also analyzed by a third method, as described by Moore et al., 2015. Here, the ryegrass samples were extracted exactly as described [35], and analyzed using an Agilent 1100 fluorescence detector (FLD). The mobile gradient and mobile phase were as described by the published method [35], except sample separation occurred on the same column used within our MS study. Excitation of ergotamine/ergovaline occurred at 310 nm, and emission was detected at 410 nm.

### 5.7. Method Application

The optimized method was applied to perennial ryegrass plants with a suite of different endophyte (Epichloë) strains (Table 5). The two-step extraction method (‘method one’) was selected based on the results from the extraction tests: the first two extracts from 20 mg of pulverized tissue were combined but not dried. The plants were extracted in triplicate and aliquoted into separate HPLC vials. Aliquots were run simultaneously on the three instruments described above. Quantitiatve results are presented as the mean value of the three samples (ppm, mg/kg).

### 5.8. Statistical Analysis

Statistical analyses including means, SD, %RSD and Student’s *t*-tests (un-paired, two tail distribution), were performed in Excel (Microsoft Office, 2010, Redmond, WA, USA). Significance was determined by *p* < 0.05.

## Figures and Tables

**Table 1:** Retention time (RT), ion mass, limit of detection (LOD), limit of quantitation (LOQ) and linear range of alkaloid standards.

Compound	RT	Ion Mass	LOD	LOQ	Standard Concentrations (ng/mL)									Equation	*R* ^2^
	(min)	(*m*/*z*)	(ng/mL)	(ng/mL)	1	2	3	4	5	6	7	8	9		
peramine	3.59	248.1501	0.2	0.8	0.8	8.0	19.9	39.8	79.7	199.2	398.5	796.9	1593.9	y = 217004x	0.9912
ergovaline/ergotamine ^1^	5.34/5.85	534.2709/582.2701	0.2	0.8	0.8	8.4	21.0	42.1	84.2	210.5	421.0	842.0	1684.0	y = 54031x	0.9998
lolitrem B	11.07	686.4037	1.8	6.0	6.0	12.0	30.0	60.0	120.0	300.0	600.0	1200.0	2400.0	y = 12113x	0.9970

^1^ Ergovaline was quantified using ergotamine. The indicated RT and mass windows for ergovaline were established using perennial ryegrass with standard toxic endophyte (E+) samples. Data was acquired on the QE MS instrument [M+H]^+^.

**Table 2:** Accuracy (bias) and precision of the alkaloid standards.

	Standard	Actual Concentration (ng/mL)	Measured Concentration (ng/mL)	Accuracy (bias) (%)	Precision (% RSD)
peramine	Low	8.0	7.9	100.2	3.5
	Med	79.7	79.2	99.7	3.4
	High	796.9	792.3	99.6	1.0
ergotamine	Low	8.4	7.9	99.1	1.4
	Med	84.2	83.2	99.4	2.4
	High	842.0	843.9	99.9	2.6
lolitrem B	Low	12.0	11.9	99.3	2.3
	Med	120.0	119.3	99.5	3.1
	High	1200.0	1194.8	100.0	2.8

Data was acquired on the QE MS instrument using duplicate analyses over eight injections.

**Table 3:** Comparison of two extraction methods showing the concentration of peramine, ergovaline and lolitrem B from perennial ryegrass with standard toxic endophyte (E+).

	Peramine				Ergovaline			Lolitrem B		
	Extract	Mean ± SD	RSD (%)	Σ	Mean Conc. (ng/mL)	RSD (%)	Σ	Mean Conc. (ng/mL)	RSD (%)	Σ
Method 1	1	32.2 ± 0.1	0.4	39.5	0.1 ± 0.01	2.2	0.1	13.6 ± 0.2	1.2	15.3
	2	7.3 ± 0.2	3.3	-	-	-	-	1.7 ± 0.1 *	4.2	-
	3	2.8 ± 0.1 *	5.0	-	-	-	-	0.2 ± 0.02 *	7.5	-
	4	1.2 ± 0.1 *	5.6	-	-	-	-	-	-	-
	5	0.6 ± 0.1 *	8.3	-	-	-	-	-	-	-
Method 2	1 & 2	33.2 ± 0.1	0.4	-	0.1 ± 0.003	2.0	-	14.3 ± 0.8	5.4	-

Method 1: samples (*n* = 5) extracted five times each and analyzed separately. Method 2: samples (*n* = 5) extracted twice each, combined, dried and reconstituted. All alkaloids were compared to external standards (ergotamine used to measure ergovaline). Mean concentrations of alkaloids and sum (Σ) of the first two extracts (method 1) are described as parts per million (ppm, mg/kg). SD, standard deviation; %RSD, percent relative standard deviation; * below the limit of quantitation (LOQ); -, not applicable. Data was acquired from the QE MS instrument.

**Table 4:** Recovery (RE) rates and matrix effect (ME) (%) of alkaloid standards spiked to three levels in perennial ryegrass quality control (QC) samples with (E+) and without (E−) endophyte.

QC Sample	Spike ^1^	Peramine		Ergotamine		Lolitrem B	
		RE	ME	RE	ME	RE	ME
E−	low	77	66	85	76	87	84
	med	79	70	87	77	86	80
	high	81	81	85	76	80	71
E+	low	88	61	85	74	84	88
	med	85	67	87	74	90	78
	high	83	67	86	73	86	67

^1^ Low, medium and high spikes are: peramine at 8.0, 79.7 and 796.9 ng/mL; ergotamine at 8.4, 84.2 and 842.0 ng/mL; and lolitrem B at 12, 120 and 1200 ng/mL. Data was acquired from the QE MS instrument.

**Table 5:** List of glasshouse-grown perennial ryegrass-endophyte associations used for the quantitation of alkaloids in this study.

Cultivar/Variety	Endophyte Strain	Qualitative Alkaloid Profile	Supplier
Alto	SE	P, E, L	Barenbrug Agriseeds
Alto	NEA3	P, E	Barenbrug Agriseeds
Trojan	WE ^1^	nil	Barenbrug Agriseeds
Trojan	NEA6	P, E	Barenbrug Agriseeds
Trojan	NEA10	P, E	Barenbrug Agriseeds
Trojan	NEA11	P, E	Barenbrug Agriseeds
Trojan	NEA47	P, E	Barenbrug Agriseeds
LP534	NEA21	P, L	Barenbrug Agriseeds
Shogun ^2^	NEA2	P, E, L (low)	Barenbrug Agriseeds

^1^ WE, without endophyte (E−). ^2^ Shogun is a perennial and annual hybrid. Alkaloid profiles are: P, peramine; E, ergovaline; L, lolitrem B.

## Supplementary Materials

The following are available online at https://www.mdpi.com/2072-6651/11/11/649/s1, Figure S1: Extracted ion chromatograms (EIC) of non-spiked perennial ryegrass without endophyte (E−), or spiked samples at low concentrations (peramine, 8.0 ng/mL; ergotamine, 8.4 ng/mL and lolitrem B, 12 ng/mL. Table S1: Matrix effect (ME) and recovery (RE) data for alkaloids spiked at low, medium and high concentrations in perennial ryegrass with (E+) and without (E-) endophyte. Table S2: Comparison of mean alkaloid concentrations for peramine, ergovaline and lolitrem B in different glasshouse-grown perennial ryegrass-endophyte associations using three different analytical instruments.Table S3: Retention time (RT), ion information, limit of detection (LOD), limit of quantitation (LOQ) and linearity range of alkaloids detected by the triple quadrupole MS (QQQ MS) instrument and fluoresce detection (FLD).
