# Supplementary material for: A Simple LC–MS Method for the Quantitation of Alkaloids in Endophyte-Infected Perennial Ryegrass
Source: Toxins (Basel). 2019 Nov 7;11(11):649. doi: 10.3390/toxins11110649 (PMC6891275; doi:10.3390/toxins11110649)
Supplement: Supplementary file 1 [file toxins-11-00649-s001.pdf]

Supplementary Materials: A Simple LC–MS Method for the Quantitation of Alkaloids in Endophyte-Infected Perennial Ryegrass

Simone Vassiliadis, Aaron C. Elkins, Priyanka Reddy, Kathryn M. Guthridge, German C. Spangenberg and Simone J. Rochfort

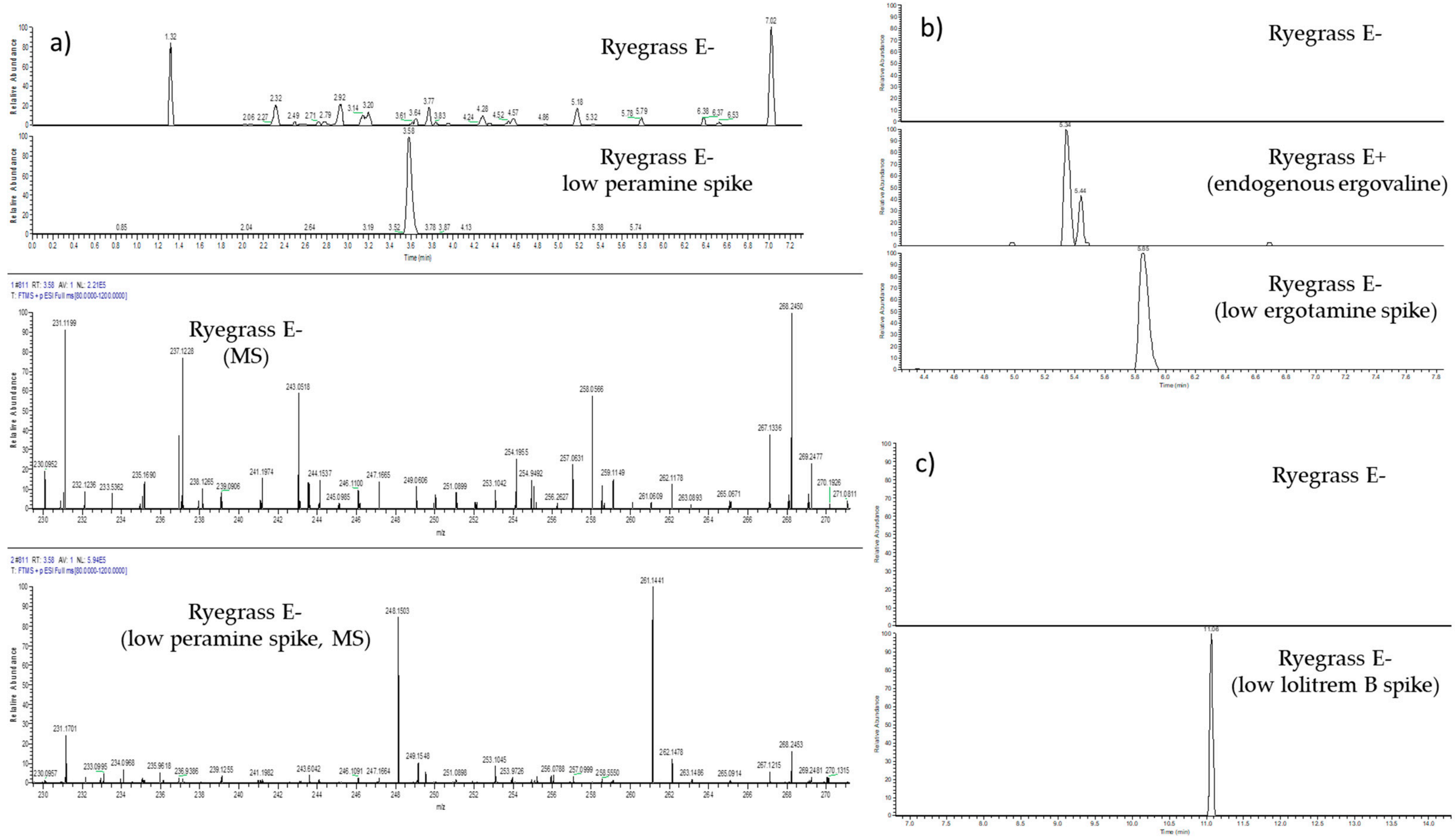

Figure S1. Extracted ion chromatograms (EIC) of non-spiked perennial ryegrass without endophyte (E−), or spiked samples at low concentrations (peramine, 8.0 ng/mL; ergotamine, 8.4 ng/mL and lolitrem B, 12 ng/mL). (**a**) The complexity of the plant matrix is demonstrated using the EIC and mass spectrum (MS) of peramine

at 3.58 min *m/z* 248.1501. (**b**) No co-eluting peaks were observed for ergovaline/ergotamine *m/z* 534.2709/582.2701 or (**c**) lolitrem B *m/z* 686.4037. Perennial ryegrass with standard toxic endophyte (E+, endogenous, no spike) was used to determine the elution of ergovaline.

Table S1. Matrix effect (ME) and recovery (RE) data for alkaloids spiked at low, medium and high concentrations in perennial ryegrass with (E+) and without (E-) endophyte.

			**Peramine**				**Ergotamine**				**Lolitrem B**			
**QC sample**	**Concentration**		**Neat std.**	**Post-spike**	**Pre-spike**	**Endogenous**	**Neat std.**	**Post-spike**	**Pre-spike**	**Endogenous**	**Neat std.**	**Post-spike**	**Pre-spike**	**Endogenous**
E-	Low	Mean.	302	199	152	na	41	31	26	na	13	11	10	na
		SD.	3.06	3.63	2.57	na	1.24	0.82	1.32	na	0.45	1.00	0.32	na
		% RSD	1.20	1.83	1.69	na	3.04	2.63	4.99	na	3.45	9.14	3.42	na
	Med	Mean.	2630	1845	1461	na	416	319	279	na	148	118	101	na
		SD.	24.19	4.46	17.29	na	12.28	11.25	7.17	na	5.45	4.29	3.62	na
		% RSD	0.92	0.24	1.18	na	2.95	3.53	2.57	na	3.69	3.64	3.59	na
	High	Mean.	18402	14821	11957	na	4533	3450	2916	na	1480	1044	835	na
		SD.	105.53	67.32	41.42	na	57.74	75.45	46.21	na	54.25	35.35	21.89	na
		% RSD	0.57	0.45	0.35	na	1.27	2.19	1.58	na	3.67	3.39	2.62	na
E+	NS	Mean.	na	na	na	9373	na	na	na	na	na	na	na	201
		SD.	na	na	na	132.28	na	na	na	na	na	na	na	4.48
		% RSD	na	na	na	1.41	na	na	na	na	na	na	na	2.23
	Low	Mean.	302	9556	9534	na	41	30	26	na	13	217	215	na
		SD.	3.06	117.08	187.48	na	1.24	0.98	1.13	na	0.45	6.30	9.45	na
		% RSD	1.20	1.23	1.97	na	3.04	3.24	4.42	na	3.45	2.91	4.39	na
	Med	Mean.	2630	11145	10536	na	416	309	268	na	148	321	310	na
		SD.	24.19	469.73	290.25	na	12.28	3.96	3.11	na	5.45	10.23	5.25	na
		% RSD	0.92	4.21	2.75	na	2.95	1.28	1.16	na	3.69	3.19	1.69	na
	High	Mean.	18402	21748	19688	na	4533	3309	2836	na	1480	1198	1057	na
		SD.	105.53	410.17	186.57	na	57.74	37.67	88.03	na	54.25	52.17	70.57	na
		% RSD	0.57	1.89	0.95	na	1.27	1.14	3.10	na	3.67	4.35	6.68	na

The mean values (peak area) and standard deviations (SD.) are in arbitrary units, $\times 10^4$. % RSD, percent relative standard deviation. Low, medium and high spikes are: peramine at 8.0, 79.7 and 796.9 ng/mL; ergotamine at 8.4, 84.2 and 842.0 ng/mL; and lolitrem B at 12, 120 and 1200 ng/mL. NS, no spike (E+ samples used to measure endogenous peramine and lolitrem B). Data was acquired from the QE MS instrument.

Table S2. Comparison of mean alkaloid concentrations for peramine, ergovaline and lolitrem B (parts per million, ppm) in different glasshouse-grown perennial ryegrass-endophyte associations using three different analytical instruments. Values of significance (compared to the QE) are highlighted (*t*-test, $p < 0.05$).

		Peramine		Ergovaline			Lolitrem B	
**Cultivar-Endophyte**	**Statistic**	**QE**	**QQQ**	**QE**	**QQQ**	**FLD**	**QE**	**QQQ**
Alto-SE	mean	50.77	52.63	0.20	0.20	0.29	20.28	18.40
	SEM	0.69	3.16	0.01	0.00	0.04	0.55	0.49
	RSD (%)	2.35	10.40	7.99	1.56	21.22	4.68	4.65
	difference (%)	-	3.65	-	-0.91	48.01	-	-9.28
	P value	-	0.60	-	0.86	0.07	-	0.06
Alto-NEA3	mean	7.84	7.93	0.29	0.29	0.29	n.d	n.d
	SEM	0.08	0.43	0.02	0.01	0.02	-	-
	RSD (%)	1.86	9.31	9.04	3.25	11.74	-	-
	difference (%)	-	1.06	-	-0.40	0.85	-	-
	P value	-	0.86	-	0.95	0.96	-	-
Trojan-WE	mean	n.d	n.d	n.d	n.d	n.d	n.d	n.d
	SEM	-	-	-	-	-	-	-
	RSD (%)	-	-	-	-	-	-	-
	difference (%)	-	-	-	-	-	-	-
	P value	-	-	-	-	-	-	-
Trojan-NEA6	mean	4.59	4.65	1.05	0.99	1.11	n.d	n.d
	SEM	0.01	0.15	0.06	0.04	0.10	-	-
	RSD (%)	0.44	5.55	10.02	6.77	15.18	-	-
	difference (%)	-	1.24	-	-6.05	12.15	-	-
	P value	-	0.72	-	0.43	0.65	-	-
Trojan-NEA10	mean	9.05	9.76	4.74	4.86	4.38	n.d	n.d
	SEM	0.04	0.67	0.16	0.28	0.19	-	-
	RSD (%)	0.74	11.93	5.92	9.79	7.46	-	-
	difference (%)	-	7.80	-	2.55	-10.05	-	-
	P value	-	0.35	-	0.72	0.21	-	-
Trojan-NEA11	mean	10.12	9.53	0.06	0.08	na	n.d	n.d
	SEM	0.16	0.81	0.01	0.00	na	-	-
	RSD (%)	2.80	14.66	16.95	6.66	na	-	-
	difference (%)	-	-5.84	-	41.03	n.a	-	-
	P value	-	0.51	-	0.02	n.a	-	-
Trojan-NEA47	mean	11.55	12.29	0.39	0.39	0.18	n.d	n.d
	SEM	0.32	0.19	0.01	0.00	0.01	-	-
	RSD (%)	4.86	2.63	6.49	1.40	10.87	-	-
	difference (%)	-	6.47	-	-1.16	-52.68	-	-
	P value	-	0.12	-	0.78	0.0004	-	-

Table S3. Continued.

		Peramine		Ergovaline			Lolitrem B	
**Cultivar-Endophyte**	**Statistic**	**QE**	**QQQ**	**QE**	**QQQ**	**FLD**	**QE**	**QQQ**
Lp534-NEA21	mean	7.07	6.65	n.d	n.d	n.d	n.d	n.d
	SEM	0.05	0.43	-	-	-	-	-

	RSD (%)	1.23	11.18	-	-	-	-	-
	difference (%)	-	-5.87	-	-	-	-	-
	P value	-	0.39	-	-	-	-	-
Shogun-NEA2	mean	1.10	0.59	n.d	n.d	n.d	1.45	1.37
	SEM	0.02	0.04	-	-	-	0.04	0.03
	RSD (%)	3.36	13.00	-	-	-	5.33	3.45
	difference (%)	-	-46.72	-	-	-	-	-5.57
	P value	-	0.0005	-	-	-	-	0.20

SEM, standard error of the mean; RSD, relative standard deviation of triplicates; n.d, not detected. The difference (%) compares the triple quadruple (QQQ) or the fluorescent detection (FLD) data with that of the Q Exactive (QE) data. No alkaloids were detected in endophyte-free plants (Trojan-WE).

Table S3. Retention time (RT), ion information, limit of detection (LOD), limit of quantitation (LOQ) and linearity range of alkaloids detected by the triple quadrupole MS (QQQ MS) instrument and fluoresce detection (FLD).

Instrument	Compound	RT (min)	Ion Mass (*m*/*z*)	Quantifier Ion (*m*/*z*)	Qualifier Ions (*m*/*z*)	LOD (ng/mL)	LOQ (ng/mL)	Linearity Range (ng/mL)	Equation	R^2
QQQ	peramine [1]	3.53	248.2	175.0	189.1	0.2	0.8	0.8–1593.9	$y=44482.5x^{0.7}$	0.9987
	ergotamine [2]	5.75	582.3	223.1	208.1	0.2	0.8	0.8–1684.0	y=3903.4x	0.9998
	ergovaline	5.23	534.3	223.1	208.1	-	-	-	-	-
	lolitrem B	11.15	686.4	628.4	238.3	1.8	6.0	6.0–2400.0	y=92.7x	0.9942
FLD	ergovaline/ergotamine	5.27/6.15	-	-	-	2.4	8.0	8.0–210.0	y=0.1629x	0.9995

[1] The standards used for peramine were not linear on the QQQ MS, therefore, a power curve was used to calculate the R^2 value. [2] Ergovaline was quantified using ergotamine. The indicated RT and mass window for ergovaline was established using perennial ryegrass with standard toxic endophyte QC samples (E+).
